# Bayesian power equivalence in latent growth curve models

**DOI:** 10.1111/bmsp.12193

**Published:** 2019-11-05

**Authors:** Angelika M. Stefan, Timo von Oertzen

**Affiliations:** ^1^ Department of Psychology University of Amsterdam The Netherlands; ^2^ Department of Psychology University of the Bundeswehr Germany

**Keywords:** Bayes factor, design analysis, power analysis, sample size, structural equation modelling

## Abstract

Longitudinal studies are the gold standard for research on time‐dependent phenomena in the social sciences. However, they often entail high costs due to multiple measurement occasions and a long overall study duration. It is therefore useful to optimize these design factors while maintaining a high informativeness of the design. Von Oertzen and Brandmaier (2013,*Psychology and Aging*, 28, 414) applied power equivalence to show that Latent Growth Curve Models (LGCMs) with different design factors can have the same power for likelihood‐ratio tests on the latent structure. In this paper, we show that the notion of power equivalence can be extended to Bayesian hypothesis tests of the latent structure constants. Specifically, we show that the results of a Bayes factor design analysis (BFDA; Schönbrodt & Wagenmakers (2018,*Psychonomic Bulletin and Review*, *25*, 128) of two power equivalent LGCMs are equivalent. This will be useful for researchers who aim to plan for compelling evidence instead of frequentist power and provides a contribution towards more efficient procedures for BFDA.

## 1 Introduction

Researchers design experiments to gain knowledge of the world. In a world of limited resources, it is ethical to conduct these experiments efficiently (Halpern *et al.*, 2002). Hunter and Hoff (1967) define research efficiency as ‘the amount of useful information obtained per unit cost’. Often, longitudinal studies entail especially high costs. These accrue either due to a long overall study duration, for example when a treatment has to be administered over a long period of time, or due to a large number of measurement occasions, for example when non‐reusable testing material is spent at each testing event. It is therefore especially important to plan longitudinal studies carefully so that an optimal balance between study costs and the expected gain in information can be achieved (Brandmaier *et al.*, 2015).

**Figure 1:**
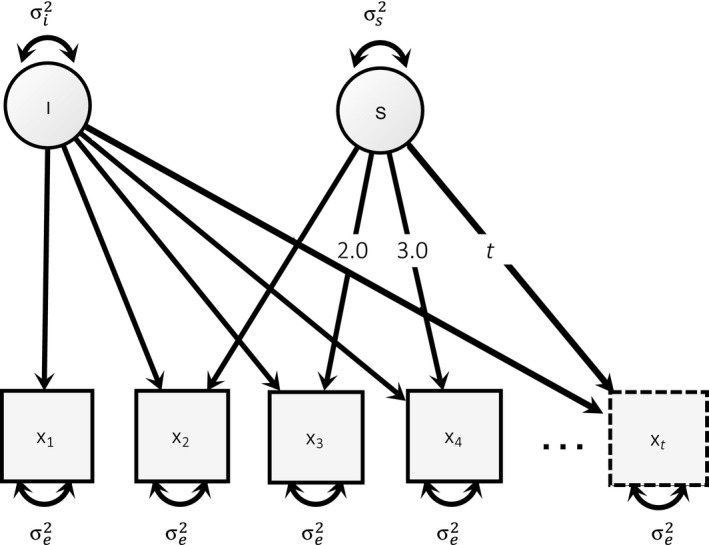
Schematic representation of a Latent Growth Curve Model (LGCM). More measurement occasions can be added as depicted for *x*_t_. Latent variables represent the intercept (*I*) with variance $\sigma_{I}^{2}$ and slope (*S*) with variance $\sigma_{S}^{2}$. Figure available under a CC‐BY4.0 license at https://osf.io/hkt4p/.

Longitudinal designs can be statistically evaluated with a sub‐group of structural equation models (SEMs; for an overview see e.g., Baltes *et al.*, 1988) called Latent Growth Curve Models (LGCMs; see e.g., Duncan & Duncan, 2009). In a simple LGCM, the values of a variable across several measurement occasions (*x_i_*) are modeled as a combination of a latent intercept (*I*) and a latent slope (*S*). The intercept has a constant influence on the measurement occasions, while the slope adds time‐dependent linear changes (see Figure 1). To add nonlinear changes, a quadratic or higher‐order term can be introduced (Duncan & Duncan,^2009^). For example, Lindenberger and Ghisletta (2009) investigated cognitive and sensory decline in elderly participants with an LGCM. In this context, the intercept parameter captured the participants' initial abilities and the slope parameter captured the extent of the linear time‐dependent decline.

An advantage of LGCMs is that they allow the direct estimation of between‐subjects variability in the latent intercept and slope, described as the variance of the intercept ( $\sigma_{I}^{2}$) and the variance of the slope ( $\sigma_{S}^{2}$) in the model. These random effects represent the individual differences in initial performance and change, respectively (Rogosa & Willett, 1985). In an LGCM where the intercept reflects the initial status of the observed variable, the intercept‐slope covariance (σ_IS_) reflects the extent to which individual differences in the initial status correlate with subsequent change (Rovine & Molenaar, 1999). Thus, in the example used earlier (Lindenberger & Ghisletta, 2009), the variance of the intercept can be interpreted as the variability of cognitive and sensory abilities of participants at the beginning of the study. The variance of the slope corresponds to differences in the steepness of the cognitive decline between participants. A positive covariance between intercept and slope in the example would show that participants with higher initial abilities suffer from a more rapid decline.

In a frequentist setting, an important aspect of the quality of a design is its statistical power, which is defined as the long‐term probability of correctly rejecting the null hypothesis under a given population effect size that differs from zero. The statistical power of a design depends on the size of the effect in the population, on the significance level α of the hypothesis test, on the sample size *N*, and on the measurement design (Brandmaier, *et al.*, 2018; Cohen, 1992). For most traditional hypothesis tests, such as a *z*‐test or a *t*‐test, it is possible to calculate the statistical power analytically (Murphy *et al.*, 2014). However, for most SEMs there is no analytical solution available, so the statistical power of a model has to be estimated via numerical approximations (e.g., Saris & Satorra, 1993) or through simulations (e.g., Hertzog, *et al.*, 2008, Muthén & Muthén, 2002).

Von Oertzen (2010) introduced the concept of power equivalence, which describes that two designs have the same statistical power to detect a true effect. Power equivalence can be used to find research designs that are most resource efficient among designs with the same power. For example, von Oertzen and Brandmaier (2013) illustrated how power equivalence facilitates finding a cost‐optimal solution among multiple longitudinal designs. In longitudinal designs, power equivalence can be established by balancing the overall duration of the study and the number of measurement occasions. To keep the power constant, more measurement occasions are required if the overall study duration is shortened. By comparing multiple power‐equivalent longitudinal designs based on data and cost estimates from the Berlin Aging Study (BASE; Ghisletta, *et al.*, 2006), von Oertzen and Brandmaier (2013) showed that the overall study costs could be reduced by 16% compared to the original design while keeping the statistical power with respect to the variance of slopes constant. Thus, power equivalence can facilitate the planning of future studies in two ways: First, instead of conducting multiple potentially resource‐intensive power analyses for different designs, a power analysis has to be computed only once for a theoretically infinite number of power‐equivalent designs. Second, knowing that certain designs do not differ in an important aspect of design quality, researchers can focus on minimizing the costs (Hunter & Hoff, 1967).

**Figure 2:**
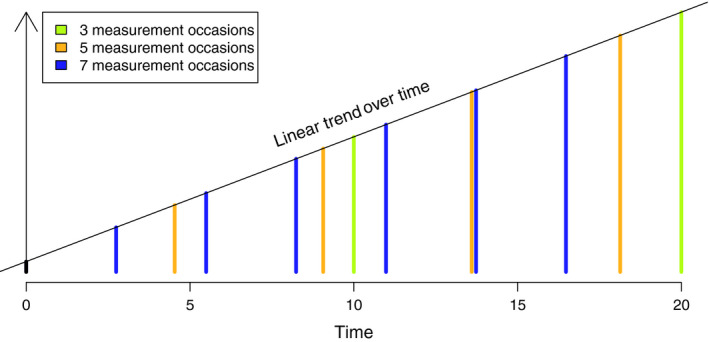
Measurement occasions of three power equivalent models measuring a linear trend. The power equivalent models were computed for $\sigma_{i}^{2}=2$, $\sigma_{e}^{2}=1$, and three, five, and seven measurement occasions. All designs assume a first measurement occasion at time *t* = 0. Figure available under a CC‐BY4.0 license at https://osf.io/hkt4p/.

Conceptually, power equivalence as applied in von Oertzen and Brandmaier (2013) can be described by the following procedure. Any LGCM can be reduced to a power equivalent model with a minimum number of observed parameters, from which further power equivalent models can be derived. These power equivalent models balance different design parameters, for example the number of measurement occasions (*j* = 1, …, *k*) and the time distance between measurement occasions, modeled in the path parameters θ*_S_* → *x_j_*, so that the power to detect an effect (e.g., σ^2^_S_ > 0) is equivalent.^1^ This is reflected in the effective error variance $\sigma_{\text{eff}}^{2}$ which is shared by all power‐equivalent models. Figure 2 schematically depicts this trade‐off: A linear trend is measured with three power equivalent designs which differ in their number of measurement occasions and their overall study duration.

In recent years, the replicability crisis (Pashler & Wagenmakers, 2012) as well as continuing criticism regarding the frequentist hypothesis testing framework (e.g., Edwards, *et al.*, 1963; Wagenmakers, 2007) have led to a growing interest in Bayesian methods for statistical inference.^2^ The single most important quantity in Bayesian hypothesis testing is the Bayes factor (Kass & Raftery, 1995). Mathematically, the Bayes factor (BF_10_) is defined as the ratio of the marginal likelihood of the data under the alternative model ( $p(D|H_{1})$ and the marginal likelihood of the data under the null model ( $p(D|H_{0})$. It provides a continuous quantification of the evidence in favor of one statistical model compared to another statistical model.

Since most researchers aim to collect compelling evidence in a study, both very large or very small Bayes factors can be regarded as a desirable outcome of a study. For example, a Bayes factor of BF_10_ = 10 indicates a tenfold increase in prior odds to posterior odds in favor of the alternative hypothesis after having observed the data, while a Bayes factor of BF_10_ = 1/10 indicates a tenfold increase in prior odds in favor of the null hypothesis. How large the Bayes factors get that an experiment yields, depends on the tested models (described by likelihoods and prior distributions), on the population effect size, on the amount of collected data, that is, the number of observations in the sample, and on the measurement design (Stefan, *et al.*, 2019). Assuming that the models are determined by the research question, only the sample size and measurement design can be directly influenced by the researcher. This shows that researchers who use Bayesian statistics to evaluate their data are also in the need to balance the costs and the information gain of their designs – in other words that design planning is an important topic from a Bayesian viewpoint, too.

How can researchers find an adequate sample size or measurement design so that their study likely yields compelling evidence, but is also designed economically? Schönbrodt & Wagenmakers (2018) proposed a framework called ‘Bayes Factor Design Analysis’ (BFDA) that enables researchers to find the expected Bayes factors of their design. Their approach is based on Monte Carlo simulations where data are repeatedly simulated under a population model (‘design prior’) and a Bayesian hypothesis test is conducted for each of these samples. BFDA is applicable to both sequential Bayesian designs, where the sample size is gradually increased until a prespecified Bayes factor is reached, and fixed‐N designs, where the sample size is specified prior to data collection. For the latter more traditional sampling procedure, a BFDA results in a distribution of Bayes factors that enables researchers to assess the informativeness of their planned design.

In this paper, we show that the notion of power equivalence can be extended to Bayesian hypothesis tests. Specifically, we show that the results of a BFDA for a fixed‐*N* design (Schönbrodt & Wagenmakers, 2018) of two power equivalent models as defined by von Oertzen (2010) are equivalent. Our findings are not only relevant on a conceptual level as they instantiate a bridge between frequentist and Bayesian methods. They also provide Bayesians with a possibility of design justification in longitudinal settings and help to save resources in design planning because computationally expensive BFDAs need to be conducted only once for power equivalent designs.

Our paper is structured as follows: First, we will formally prove the equivalence of BFDA results for power equivalent models. In a second step, we will substantiate our proof with a simulation for power equivalent LGCMs. Then, we will provide an application example that illustrates how Bayesian power equivalence can facilitate design planning. We will discuss the implications and limitations of our findings at the end of this article.

## 2 Formal proof of BFDA equivalence for power equivalent models

In this section, we show formally that two power equivalent models with the same parameter set θ will also produce the same distribution of the Bayes Factor when comparing two hypotheses about θ under data generated by a population model. We assume that both hypotheses are given by a prior distribution π_1_ and π_1_ for θ, where as usual one or both can be point hypotheses, i.e., degenerated prior distributions with the mass fixed at any specific point.

**Figure 3:**
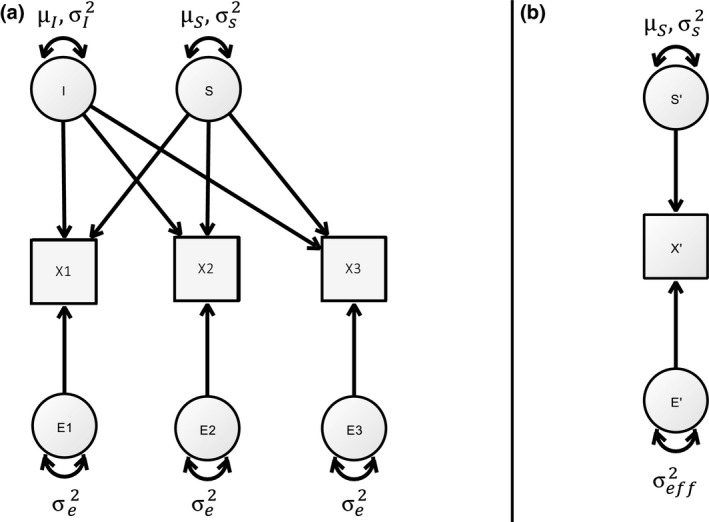
Power equivalent reduction of an LGCM. Panel A shows an LGCM with three measurement occasions which can be reduced to the minimal power equivalent model displayed in panel B. Figure available under CC‐BY4.0 license at https://osf.io/hkt4p/.

Power equivalence on multivariate normal models, as defined in von Oertzen (2010), can be expressed as a combination of two basic power equivalent operations. The first one is a linear transformation of the observed variables, the second an omission of observed variables with a probability distribution which is constant with respect to θ, and which are independent of other variables. For example, in an LGCM, the linear transformation transforms the measurement model into a minimal model with one observed variable that is dependent on the latent slope and a number of variables that are independent of the latent slope (and hence of the slope variance parameter). An example for a power equivalent transformation of an LGCM can be seen in Figure 3. The mathematical details of the calculation can be found in the Appendix.

Let (*S*,*m*) be the estimated covariance matrix and mean of a sample and ( Σ, μ) of a model. In the following, we will write $L_{\Sigma ,\mu }\left(S,m\right)$ for the minus two log likelihood, i.e.,

$$L_{\Sigma ,\mu }\left(S,m\right)=-2\log L\left(S,m|\Sigma ,\mu \right).$$

We start by showing two simple lemmas.

Lemma 1 For any multivariate normal model with covariance matrix Σ and mean μ, an orthogonal transformation *Q* on the model space does not change the likelihood function.

The minus two log likelihood of a multivariate normal with parameter μ and Σ and a dataset with mean *m* and covariance matrix *S* per participant is

$$L_{\Sigma ,\mu }\left(S,m\right)=c\;+\;\ln\left(|\Sigma |\right)\;+\;\text{Tr}\left(\Sigma^{-1}S\right)\;+\;{\left(m-\mu \right)}^{T}\Sigma^{-1}\left(m-\mu \right).$$

Transforming all four distribution parameters with *Q* results in

$$\begin{array}{c} L_{Q\Sigma Q^{T},Q\mu }\left(QSQ^{T},Qm\right)=c+\ln(|Q\Sigma Q^{T}\left|\right)+\text{Tr}\left(Q\Sigma^{-1}Q^{T}QSQ^{T}\right)\\ +{\left(m-\mu \right)}^{T}Q^{T}Q\Sigma^{-1}Q^{T}Q\left(m-\mu \right)\\ =c+\ln(|Q\Sigma Q^{T}\left|\right)+\text{Tr}\left(Q\Sigma^{-1}SQ^{T}\right)+{\left(m-\mu \right)}^{T}\Sigma^{-1}\left(m-\mu \right), \end{array}$$

where the determinant and the trace do not change by an orthogonal transformation, therefore,

$$\begin{array}{c} -2\log L\left(QSQ^{T},Qm|Q\Sigma Q^{T},Q\mu \right)=c+\ln\left(|\Sigma |\right)+\text{Tr}\left(\Sigma^{-1}S\right)+{\left(m-\mu \right)}^{T}\Sigma^{-1}\left(m-\mu \right)\\ =L_{\Sigma ,\mu }\left(S,m\right). \end{array}$$

Lemma 2 For any multivariate normal model with covariance matrix Σ and mean μ, omitting observed variables which have distributions that are constant with respect to some parameter set θ and are independent of all other parameters does not change the likelihood ratio of any two parameter values θ_1_ and θ_2_.

For simplicity of notation, we prove that the difference of the minus two log likelihoods is constant. Let $\Sigma =\left(\begin{array}{cc} \Sigma_{1}\left(\theta \right) & 0\\ 0 & \Sigma_{2} \end{array}\right)$ be the separation of Σ and $\mu =\left(\begin{array}{c} \mu_{1}\left(\theta \right)\\ \mu_{2} \end{array}\right)$ be the separation of μ into a first part that depends on θ and a second, independent part that does not. We separate the data distribution accordingly. Note that the covariances between the two blocks in the data distribution are not relevant for the likelihood, i.e., we can write

$$\begin{array}{c} L_{\Sigma (\theta ),\mu (\theta )}\left(S,m\right)=c+\ln(|\Sigma_{1}\left(\theta \right)\left|\right)+\text{Tr}\left(\Sigma_{1}{\left(\theta \right)}^{-1}S_{1}\right)+{\left(m_{1}-\mu_{1}\left(\theta \right)\right)}^{T}\Sigma_{1}{\left(\theta \right)}^{-1}\left(m_{1}-\mu_{1}\left(\theta \right)\right)\\ \;+\;\ln(|\Sigma_{2}\left|\right)+\text{Tr}\left(\Sigma_{2}^{-1}S_{2}\right)+{\left(m_{2}-\mu_{2}\right)}^{T}\Sigma_{2}^{-1}\left(m_{2}-\mu_{2}\right) \end{array}$$

When taking the difference of the minus two log likelihoods for θ_1_ and θ_2_, the second part of the equation and *c* cancels, so that the difference solves to

$$\begin{array}{c} L_{\Sigma \left(\theta_{1}\right),\mu \left(\theta_{1}\right)}\left(S,m\right)-L_{\Sigma \left(\theta_{2}\right),\mu \left(\theta_{2}\right)}\left(S,m\right)=\ln(|\Sigma_{1}\left(\theta_{1}\right)\left|\right)+\text{Tr}\left(\Sigma_{1}{\left(\theta_{1}\right)}^{-1}S_{1}\right)\\ +{\left(m_{1}-\mu_{1}\left(\theta_{1}\right)\right)}^{T}\Sigma_{1}{\left(\theta_{1}\right)}^{-1}\left(m_{1}-\mu_{1}\left(\theta_{1}\right)\right)\\ -\ln(|\Sigma_{1}\left(\theta_{2}\right)\left|\right)-\text{Tr}\left(\Sigma_{1}{\left(\theta_{2}\right)}^{-1}S_{1}\right)\\ -{\left(m_{1}-\mu_{1}\left(\theta_{2}\right)\right)}^{T}\Sigma_{1}{\left(\theta_{2}\right)}^{-1}\left(m_{1}-\mu_{1}\left(\theta_{2}\right)\right)\\ =L_{\Sigma_{1}\left(\theta_{1}\right),\mu_{1}\left(\theta_{1}\right)}\left(S_{1},m_{1}\right)-L_{\Sigma_{1}\left(\theta_{2}\right),\mu_{1}\left(\theta_{2}\right)}\left(S_{1},m_{1}\right). \end{array}$$

We conclude that the likelihood ratio remains constant under both base power equivalent operations, and hence under all combinations of those. Since the Bayes factor is the ratio of two prior‐weighted likelihoods, we conclude further that the Bayes factor is unaltered by power equivalent transformations for any data set (*S*,*m*) and parameter sets θ_1_ and θ_2_. Thus, in particular, the distribution of the Bayes factor is identical for any priors π_1_ and π_2_ and any data distribution:

Corollary 3 If $\left(\Sigma_{A}\left(\theta \right),\mu_{A}\left(\theta \right)\right)$ and $\left(\Sigma_{B}\left(\theta \right),\mu_{B}\left(\theta \right)\right)$ are two power equivalent multivariate normal models *A* and *B*, then under any distribution for data sets (*S*,*m*) and prior distributions π_1_ and π_2_ to be compared, the corresponding distribution of the Bayes factor is identical for both models.

For simplicity, we omit the explicit separation of *S* and *m* in *S*_1_ and *S*_2_ and *m*_1_ and *m*_2_, respectively, because the irrelevant parts are ignored by the likelihood function (see proof of Lemma 2). For any specific outcome (*S*,*m*) of the random variable representing the data, let (*S* ^*^, m*) be the power equivalent transformation of the data as explained at the beginning of this section. The Bayes factor for the first model is given by

$${\text{BF}}_{A_{12}}\left(S,m\right)=\frac{\int_{\theta_{1}}L\left(S,m|\Sigma_{A}\left(\theta_{1}\right),\mu_{A}\left(\theta_{1}\right)\right)\pi_{1}\left(\theta_{1}\right)d\theta_{1}}{\int_{\theta_{2}}L\left(S,m|\Sigma_{A}\left(\theta_{2}\right),\mu_{A}\left(\theta_{2}\right)\right)\pi_{2}\left(\theta_{2}\right)d\theta_{2}}$$

which can be rewritten as

$$\begin{array}{c} {\text{BF}}_{A_{12}}\left(S,m\right)=\int_{\theta_{1}}\frac{L\left(S,m|\Sigma_{A}\left(\theta_{1}\right),\mu_{A}\left(\theta_{1}\right)\right)\pi_{1}\left(\theta_{1}\right)}{\int_{\theta_{2}}L\left(S,m|\Sigma_{A}\left(\theta_{2}\right),\mu_{A}\left(\theta_{2}\right)\right)\pi_{2}\left(\theta_{2}\right)d\theta_{2}}d\theta_{1}\\ =\int_{\theta_{1}}\frac{1}{\int_{\theta_{2}}\frac{L\left(S,m|\Sigma_{A}\left(\theta_{2}\right),\mu_{A}\left(\theta_{2}\right)\right)\pi_{2}\left(\theta_{2}\right)}{L\left(S,m|\Sigma_{A}\left(\theta_{1}\right),\mu_{A}\left(\theta_{1}\right)\right)\pi_{1}\left(\theta_{1}\right)}d\theta_{2}}d\theta_{1}\\ =\int_{\theta_{1}}\frac{1}{\int_{\theta_{2}}\frac{L\left(S^{*},m^{*}|\Sigma_{B}\left(\theta_{2}\right),\mu_{B}\left(\theta_{2}\right)\right)\pi_{2}\left(\theta_{2}\right)}{L\left(S^{*},m^{*}|\Sigma_{B}\left(\theta_{1}\right),\mu_{B}\left(\theta_{1}\right)\right)\pi_{1}\left(\theta_{1}\right)}d\theta_{2}}d\theta_{1}\\ =\frac{\int_{\theta_{1}}L\left(S^{*},m^{*}|\Sigma_{B}\left(\theta_{1}\right),\mu_{B}\left(\theta_{1}\right)\right)\pi_{1}\left(\theta_{1}\right)d\theta_{1}}{\int_{\theta_{2}}L\left(S^{*},m^{*}|\Sigma_{B}\left(\theta_{2}\right),\mu_{B}\left(\theta_{2}\right)\right)\pi_{2}\left(\theta_{2}\right)d\theta_{2}}\\ ={\text{BF}}_{B_{12}}\left(S^{*},m^{*}\right). \end{array}$$

Since the Bayes factor is identical for both models for any specific outcome of the data, its distribution under any random distribution of (*S*,*m*) is identical for both power equivalent models.

## 3 Simulation study

We performed a simulation study to illustrate the equivalence of Bayes factor distributions for power equivalent LGCMs. As in von Oertzen and Brandmaier (2013), we concentrated on a single parameter of interest: $\sigma_{S}^{2}$, the interindividual variance in the latent slope parameter. The focal Bayesian hypothesis test therefore compared the two hypotheses $H_{0}$: $\sigma_{S}^{2}=0$ and $H_{1}:\sigma_{S}^{2}∼\pi_{1}$ where π_1_, is a prior distribution that allows the parameter $\sigma_{S}^{2}$ to vary. We operationalized this prior distribution as a gamma distribution with a shape parameter of *k* = 1 and a rate parameter of β = 0.5. This prior places most weight on parameter values between 0 and 6 and can be considered as an example for an informed prior for typical effect sizes in psychology (see e.g., Duncan, *et al.*, 2006; Iddekinge, *et al.*, 2009; von Oertzen & Brandmaier, 2013). In this special case, all parameters of the model apart from $\sigma_{S}^{2}$ are considered to be known and fixed. Thus, the Bayes factor can be calculated through a simple integration procedure.

For our simulation study, we conducted a total of 36 BFDAs, where each BFDA result is based on 1000 Bayes factors. All BFDAs were performed using the following Monte Carlo simulation algorithm:

1. Find three power equivalent models with the given parameters for $\sigma_{E}^{2}$ and $\sigma_{I}^{2}$;
2. simulate 1,000 datasets for each of the models given a certain population parameter (design prior) for $\sigma_{S}^{2}$;
3. compute the Bayes factor for each of the datasets.

We compare the results of a fixed‐N BFDA for 3 power equivalent LGCMs under 12 different population models (design priors). The three power equivalent models have 7, 5, and 3 equally distanced measurement occasions, respectively, and were computed using the equations provided in von Oertzen and Brandmaier ( 2013; see the Appendix below). In the simulations, we varied the variance of the intercept $\sigma_{I}^{2}$, the residual variance $\sigma_{E}^{2}$, and the true variance of the slope ( $\sigma_{S}^{2}$ | *H*1). All BFDAs were conducted for a sample size of *N* = 300.

**Figure 4:**
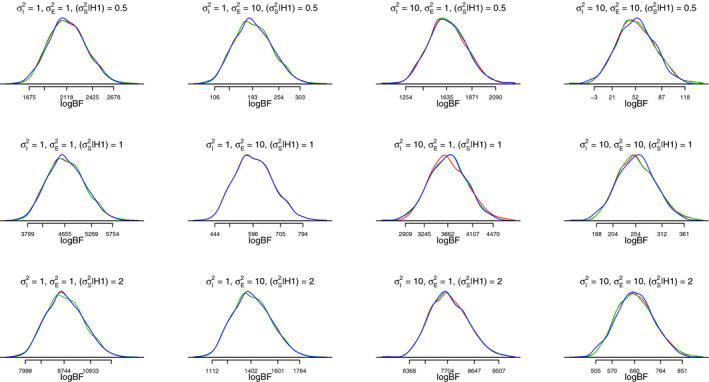
BFDAs for power equivalent models yield almost identical results. Figure shows the distribution of the log Bayes factors for power equivalent models with 3 (colored red), 5 (colored green), and 7 (colored blue) measurement occasions. Simulations were conducted with 1,000 iterations and different population parameters for the variance of the intercept $\sigma_{i}^{2}$, error variance $\sigma_{e}^{2}$, and variance of the slope $\sigma_{S}^{2}$ on a fixed sample size of *N* = 300. Figure available under a CC‐BY4.0 license at https://osf.io/hkt4p/.

Figure 4 shows the distributions of log Bayes factors for the three power equivalent models under all simulated conditions. Overall, the distributions are nearly identical for the power equivalent models which illustrates the formal proof of BFDA equivalence conducted in the previous section of this article. Generally, the Bayes factors are very large, which happens due to the relatively large dataset and the assumption that several important parameter values of the model are already known. There are small differences in the Bayes factor distributions that can be explained through the random variation in the simulation process.

The simulation code as well as the simulation results are openly accessible on https://osf.io/hkt4p/.

## 4 Application example: Effects of a mindfulness training

In this section, an applied example is discussed that illustrates how the notion of power equivalence can be used to facilitate *a‐priori* design analyses for longitudinal studies. We will build on a study by Kiken *et al*(2015) who investigated the psychological effects of a mindfulness training. Mindfulness is a cognitive state of nonjudgmental awareness in which an individual pays attention to the thoughts, emotions, and sensations of the moment. Kiken *et al*(2015) measured state mindfulness with the Toronto Mindfulness Scale (Lau *et al.*,^2006^) at seven equally distanced measurement occasions during an ongoing mindfulness training that was directed at increasing the participants' general level of mindfulness. Using an LGCM, they concluded that while the training led on average to an increase in mindfulness, there were noticeable differences between individuals regarding the amount of change, i.e., there was considerable variability in the slope of state mindfulness.

In this example application, we assume that researchers developed a new training method that is supposed to be equally effective for all participants. As the researchers would like to quantify evidence in favor of the null hypothesis ( $\sigma_{S}^{2}=0$), they decide to use Bayesian hypothesis testing (Wagenmakers *et al*, 2018). When planning the study, they have two goals: Making sure that their envisioned sample size is large enough to obtain strong evidence in favor of the null hypothesis ( ${\text{BF}}_{01}\geq 10$) if the null hypothesis is true and minimizing the overall study costs. For this example, we roughly estimate that the costs for each measurement occasion are $10 per participant (e.g., for participant compensation or data entry), and that the running costs are $500 per week (e.g., for renting lab space, employing assistants to run the study). We further assume that the envisioned sample size of the researchers is *N* = 50. Thus, when planning the study, two design questions come up: (1) Is a sample size of *N* = 50 enough to achieve strong evidence in favor of the null hypothesis when the null hypothesis is true, and (2) which of the power equivalent designs is most cost‐efficient?

**Figure 5:**
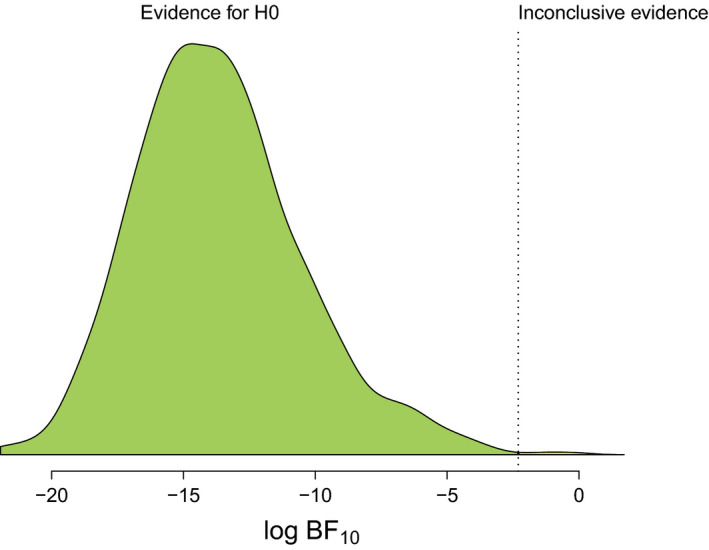
Bayes factor distribution resulting from a BFDA based on the results of Kiken et al. (2015) for a design with a fixed sample size of *N* = 50 and a true population effect size of $\sigma_{S}^{2}=0$. Figure available under a CC‐BY4.0 license at https://osf.io/hkt4p/.

First, the researchers can now conduct a BFDA based on the design and results of the original study, that is seven equally distanced measurement occasions, a variance of intercepts of $\sigma_{I}^{2}=43.6$, and an error variance of $\sigma_{E}^{2}=21.45$. The results for a sample size of *N* = 50 show that the Bayes factor $\left({\text{BF}}_{10}\right)$ will be smaller than 0.1 in 99.8% of the cases, that is, there is a high chance to obtain strong evidence in favor of the null hypothesis if the null hypothesis is true (see Figure 5). Being convinced by the high degree of informativeness, the researchers can now proceed to find the most cost‐efficient design with the same power. Using power equivalence, the researchers can come up with several power‐equivalent measurement designs. Table 1 shows three power‐equivalent designs with 3, 7, and 10 measurement occasions, respectively (see the Appendix for details about the computation). All these designs share the same Bayes factor distribution based on the BFDA of the original design. However, they differ in their respective costs. As we can see from the total costs in Table 1, the measurement design with three measurement occasions is the most cost‐efficient. Prolonging the overall study duration by 1.2 weeks, but reducing the number of measurement occasions to three can therefore lead to a cost reduction of roughly 20%. There is no need to recalculate the BFDA because the researchers already know that all power‐equivalent designs are equally informative.

**Table 1:** Power‐equivalent models for testing the variance of slopes in a mindfulness training based on results of Kiken *et al.*(2015)

Waves	Assessment time	Wave costs	Running costs	Total costs
3	7.28	1,500	3,642	5,142
7	6.00	3,500	3,000	6,500
10	3.55	5,000	1,773	6,773

## 5 Discussion

Reducing study costs while keeping the results informative is an important practical objective of experimental design (Hunter & Hoff, 1967). In longitudinal studies, a cost reduction can often be achieved by finding a trade‐off between the total duration of the study and the number of measurement occasions. In a frequentist setting, researchers can optimize this trade‐off while keeping the design informative by comparing several power equivalent models (von Oertzen, 2010; von Oertzen & Brandmaier, 2013). While these models all have the same statistical power (Cohen, 1992), they exhibit different combinations of overall study length and number of measurement occasions. In this paper, we showed that the notion of power equivalence can be transferred to a Bayesian hypothesis testing framework. Specifically, we could show that power equivalence models yield the same Bayes factor distributions in a Bayes Factor Design Analysis (BFDA; Schönbrodt & Wagenmakers 2018). Therefore, power equivalent designs are equally informative both from a frequentist and Bayesian viewpoint. This shows that power equivalent models can also be used in Bayesian design planning to negotiate trade‐offs between costs and informativeness in longitudinal studies.

Our findings can be interpreted as an extension of both power equivalence (von Oertzen & Brandmaier, 2013; von Oertzen, 2010) and BFDA (Schönbrodt & Wagenmakers, 2018). From the perspective of power equivalence, we provide a straightforward generalization of the approach and show that it can also be used in the Bayesian process design planning. This highlights the relevance of the approach and raises the question whether further generalizations are possible. For example, the general notion of power equivalence could be generalized to statistical models other than Latent Growth Curve Models (LGCMs). Our results show that this would be a relevant contribution to design planning methods both from a frequentist and a Bayesian viewpoint. From the perspective of BFDA, our findings provide a first step towards a simplification of the procedure. Since the approach is based on Monte Carlo simulations, conducting a BFDA can be computationally expensive. Finding models that yield the same BFDA results can substantially facilitate the process of Bayesian design planning because a BFDA needs to be conducted only once for all of these power equivalent models. Our results show that finding such power equivalent models is possible. Future research could be directed at finding more conditions for equality of BFDA results and to extend our results to sequential Bayesian designs.

By making power equivalence available to a new statistical domain, our study increases its practical applicability to the planning of experimental designs. Additionally, we make it easy for researchers to optimize their study designs based on power equivalence and BFDA by providing the code for all analyses conducted in this paper online (see https://osf.io/hkt4p/). By using well‐documented functions, we hope to encourage researchers to reuse our code and adapt it to their own practical applications. However, currently, the practical applicability of power equivalence in experimental design is still restricted by two important limitations. Firstly, the mathematical derivation for power equivalence requires that parameters which are not part of the hypothesis (in our example the variance of the intercept $\sigma_{I}^{2}$ and the error variance $\sigma_{E}^{2}$) are fixed. In practice, this is a strong assumption. However, if these parameters are not known, they (or the effective error $\sigma_{\text{eff}}^{2}$) can be estimated prior to the computation of power equivalence. A second limitation is that currently power equivalence requires a fixed structure matrix (von Oertzen, 2010), so it is only directly applicable to models like LGCMs, Change Score Models, Dual Change Score Models, Latent Differential Models, and basic models (e.g., ANOVAs). Nevertheless, these describe a considerable part of SEMs used today.

From a broader perspective, our findings illustrate that despite of methodological differences and occasional heated debates between frequentist and Bayesian methods and their respective proponents (see e.g., Wagenmakers, *et al.*, 2008), often relevant insights can be gained from describing the world from both perspectives. We hope that by showing how the notion of power equivalence and the BFDA method can be combined, we will have made a contribution towards an increased feasibility of Bayesian experimental planning. Eventually, we hope that the existence of straightforward methods for design planning can encourage more researchers to plan their study designs for efficiency and informativeness.

## Acknowledgements

We want to thank Andreas Brandmaier and Eric‐Jan Wagenmakers for their comments on an earlier draft of this paper. This research was supported by an NWO grant to AS (406.18.556).

6

## Footnotes

1 Note that although *j* in theory can go down to *j* = 1, in practical cases *j* needs to be at least equal to the number of latent variables (e.g., two for a linear LGCM, or three for a quadratic LGCM) to estimate all distribution parameters of the latent variables.

2 An easily accessible introduction to Bayesian inference can be found in Etz & Vanderkerckhove (2018).

## Appendix 1

### Computation of power equivalent models

Equations for the computation of power equivalent LGCMs following von Oertzen and Brandmaier (2013). For any original model with measurement occasions at *t*_old_, we can construct a power equivalent model with $\tilde{n}$ measurement occasions at *t*_new_.

(1) $$t_{\text{new}}=\lambda \cdot \tilde{t},$$

where

(2) $$\begin{array}{c} \lambda =\sqrt{\frac{\sigma_{E}^{2}}{\sigma_{\text{eff}}^{2}}\cdot \frac{\sigma_{I}^{2}\tilde{n}+\sigma_{E}^{2}}{\left(\sigma_{I}^{2}\tilde{n}+\sigma_{E}^{2}\right)\sum_{j=1}^{\tilde{n}}{\tilde{t}}_{j}^{2}-\sigma_{I}^{2}{\left(\sum_{j=1}^{\tilde{n}}\tilde{t}\right)}^{2}}},\\ {\tilde{t}}_{j}=\left(j-1\right)\cdot \frac{\max(t_{\text{old}})}{\tilde{n}-1},\\ \sigma_{\text{eff}}^{2}=\frac{\sigma_{E}^{2}\left(\sigma_{I}^{2}\tilde{n}+\sigma_{E}^{2}\right)}{\left(\sigma_{I}^{2}\tilde{n}+\sigma_{E}^{2}\right)\sum_{j=1}^{\tilde{n}}{\left(\lambda \tilde{t_{j}}\right)}^{2}-\sigma_{I}^{2}{\left(\sum_{j=1}^{\tilde{n}}{\tilde{t}}_{j}\right)}^{2}}. \end{array}$$

The residual variance $\sigma_{E}^{2}$ and the intercept variance $\sigma_{I}^{2}$ are considered to be known and fixed and do not differ between models.

In our simulations, we used power equivalent models with 7, 5, and 3 measurement occasions. The power equivalent models with 5 and 3 measurement occasions were derived from the model with 7 measurement occasions at *t* = 0, 1,..., 6.

In our application example, we used a design with 7 measurement occasions at *t* = 0, 1,..., 6 as a starting point and derived power equivalent models with 3 and 10 measurement occasions from this model using the equations above. The variance of the intercept $\sigma_{I}^{2}$ and the error variance $\sigma_{E}^{2}$ were derived from Kiken, *et al.*(2015) and were set to $\sigma_{I}^{2}=43.6$ and $\sigma_{E}^{2}=21.45$.
